# Obituary Notice

**Published:** 1834

**Authors:** 


### Art. VI. Obituary Notice.

*Dr. Sanders.**—Among the numerous victims who have fallen beneath the pestilence that lately passed through our country, spreading wo and desolation in its progress, perhaps none is more deeply lamented than *Thomas Sanders, M. D.*, who departed this life at his residence in Shelbyville, Ky. on the 21st of July, 1833, aged 39 years. It is a sad and painful task to record the untimely death of an early and affectionate friend, but the ties which united us whilst living, and a custom, founded upon the benevolent and sacred feelings of the human heart, call for this humble tribute to his memory. When the silent tomb closes over objects of the most tender regard and cherished affection, there is no consolation left us, but to dwell with melancholy pleasure upon their memories.

The subject of these remarks, received the honours of Transylvania University, in the year 1824. Inquisitive, ardent, and aspiring, he located himself in Shelbyville, and commenced his professional career. Endowed with intellect of a superior order, and stimulated by a spirit of the most laudable ambtion, he embraced his profession with pride, and pursued it with the eagerness of a true lover of knowledge. The narrow and circumscribed paths of the science could not confine him. He aimed at the highest walks of the profession. He regarded the science of medicine as a science of the noblest order, fraught with the destiny of millions, and capable of dispensing blessings or heaping curses upon the human race. With this enlarged view of the profession, he strove to ameliorate the condition of all about him. The subtleties that lie concealed in the labyrinth of medical lore, the revelations of nature and experience, and the developments of science, were suoght after and mastered, with the eager and untiring spirit of a philanthropist. But in the prime and vigor of manhood, upon the threshold of professional usefulness, and just beginning to reap the harvest of his early devotions, he was suddenly arrested in his earthly pilgrimage, and cut off from accomplishing the task he had so nobly begun.

* From a Correspondent.

In the death of Dr. Sanders the society where he lived is bereaved of a highly valued and much esteemed member, and the Faculty has lost one of its brightest ornaments.

